# Plant Growth and Fruit Nutrient Changes in *Carica papaya* L. Genotypes Subjected to Regulated Deficit Irrigation

**DOI:** 10.3390/life12111831

**Published:** 2022-11-09

**Authors:** Jalel Mahouachi, Elías Marrero-Díaz

**Affiliations:** Departamento de Ingeniería Agraria y del Medio Natural, Universidad de La Laguna, Ctra. de Geneto, 2, E-38200 La Laguna, Spain

**Keywords:** drought, fruit development, total soluble solutes

## Abstract

The current genotypes of *Carica papaya* L. cultivated worldwide are considered relatively tolerant or sensitive to drought conditions, depending specifically on the cultivar features and the severity of water shortage. In this work an experimental field was established by subjecting “Intenzza” and “Siluet” to the following water regimes: Control (“CT”), plants irrigated at field capacity (100%); Moderate Deficit Irrigation (“MDI”, watered at 66%); and Severe Deficit Irrigation (“SDI”, watered at 50%). The results indicated that water deficit decreases leaf number leading to a decline of total leaf area, being “Intenzza” equally affected by “SDI” and “MDI”, whereas in “Siluet” the major decrease was induced by “SDI”. Regarding fruit development, in “Intenzza”, “MDI” and “SDI” did not affect fruit number except two dates (120 and 202 DAT), decreased fruit fresh weight (FW), and increased total soluble solutes (TSS) levels, while in “Siluet” only “SDI” reduced fruit FW and watering systems did not change TSS, suggesting a better performance of this cultivar under “MDI”. In addition, drought did not impair fruit mineral concentrations excepting in few dates, and in such cases stressed fruits accumulated a slight minor or even major concentration of some nutrients than control, maintaining consequently suitable organoleptic quality.

## 1. Introduction

*Carica papaya* L. are classified as herbaceous although their heights can reach 9 to 10 m in tropical climates, and due to such stature, they are defined as giant herbs [1]. Papaya plants are generally considered susceptible to drought and responsive to irrigation [2]. Therefore, to improve growth and fruit production, irrigation supply is recommended [1]; however, inappropriate water management can negatively alter various physiological processes [3,4].

Under drought conditions, papaya plants tend to optimize water use efficiency and translocate nutrients to the youngest leaves through the removal of the oldest ones to reduce transpiration rate and maintain persisted leaves turgor [5,6]. It has been reported that water deficit (30%) in both partial root drying and regulated deficit irrigation treatments does not reduce vegetative growth or yield expressed as fruit number and fruit weight per plant compared to full irrigation [7].

Regarding flowering, papaya plants possess three sexual forms (hermaphrodite, female, and male), being the hermaphrodite and female as single flowers, and the male as large clusters [8]. This characteristic of trioecy has been reported in papaya and other species [9], and appear to be derived from a dioecious ancestor by the loss of carpel suppressor function [10]. In general, papaya continuously produce flowers and fruits after flowering initiation and leaves senesce and abscise before fruits are harvested [11]. The fruits derived from hermaphrodite flowers are the most required by the consumer as fresh fruits.

Water-deficit priming in papaya increases stomatal conductance and photosynthetic rate; however, such responses are transitory and do not enhance tolerance to subsequent severe water deficit [12,13]. At photochemical level, the application of priming in papaya induces a decrease of absorption and dissipation, and an increase of electron transport through the photosystem I [12].

Defoliation experiments show that the removal of 75% of the available leaves per plant reduces the number of new flowers, fruit set and fruit total soluble solids (TSS) at ripening, while the elimination of 50% of the leaves does not affect those variables. In addition, the natural and continuous abscission of old leaves decrease fruit set, fruit weight, and TSS [11]. The senescence of these old leaves aggravated by water stress could reduce the photosynthetic pigments since chlorophylls may reflect the photosynthetic capacity of the plants [14,15], and carotenoids have been as well involved against oxidative stress through their antioxidant activities [16,17,18].

From the physiological and nutritional perspective, water deficit can induce common and dissimilar effects in fruit crop species. It has been reported that the TSS content increase in fruits of apple trees subjected to long-term deficit irrigation (LTDI) and middle-term deficit irrigation (MTDI); however, fructose, sorbitol, total soluble sugar, and K^+^ levels are higher in fruits produced in plants treated by LTDI than in control and MTDI. In addition, both water stress limitations reduce trunk thickness, photosynthetic rate, stomatal conductance, and leaf water potential in comparison to full-irrigated trees; whilst leaf area and shoot length only decrease in stressed trees (LTDI) [19]. An experimental system studying the effects of deficit irrigation on vine mineral nutrition and growth indicates that water deficit applied before fruit set decreases the cluster size and the number of berries, mostly under nitrogen scarcity [20]. Moreover, the regulated deficit irrigation combined with low to moderate rates of nitrogen supply between bloom and veraison reduce canopy, berry size, yield, accelerate ripening, and improve fruit color [20]. In tomato plants, water stress declines the uptake of N, K^+^, Ca^2+^, Mg^2+^, Na^+^, and increases glucose, fructose, sucrose, and acid concentrations (ascorbic, malic and citric acid) in the fruits [21].

Currently, the scarcity of fresh water as a natural resource especially in arid and semiarid regions is exacerbated by the climate change, the lowering of precipitation and the increase of water demand by the population for domestic, tourism, and agricultural uses. The negative impact of drought on crops is well-known especially for those cultivated under irrigation as formerly reported. Particularly, the requirement of irrigation water for *Carica papaya* production is very high to obtain an adequate yield and may depend on genotype features. Thus, the improvement of irrigation system management could help plants to maintain rational productivity and reduce water supply. This work was performed to investigate how papaya cultivars can respond to deficit irrigation through the study of various parameters linked to plant and fruit growth, and fruit nutrient concentrations. These responses could reveal the mechanism of papaya adaptation to drought and the effectiveness of the strategy of regulated deficit irrigation system for saving water and increasing water use efficiency.

## 2. Materials and Methods

### 2.1. Plant Materials and Experimental Conditions

To assess the effects of water deficit regimes on plant growth and fruit development characteristics in *Carica papaya* cultivars, “Intenzza” and “Siluet” were used due to their great productivity and organoleptic properties at least under tropical conditions (semillasdelcaribe.com.mx, accessed on 29 September 2022). “Intenzza” has a high plant vigor with elevated yield (100–150 t ha^−1^), single fruit weight (1.5–2.0 kg), and TSS (10–13 °Brix); however, “Siluet” has a moderate plant vigor with average yield (70–90 t ha^−1^), single fruit weight (1–1.5 kg), and TSS (13–15 °Brix) as reported by *Semillas del Caribe* (semillasdelcaribe.com.mx, accessed on 29 September 2022).

Papaya seedlings (30 cm long) provided by *Cuplamol* nursery S.L. were transplanted in experimental fields under greenhouse (6–7 m in height) using a full 40-mesh cover at Tejina location ((28°31′41″ N; 16°23′13″ W; 111 m altitude), Tenerife, Canary Islands, Spain). As suggested by the nursery, at the beginning four plants were transplanted per hole and at the onset of flowering only one hermaphroditic plant was kept for continuing the experiments and the rest of the plants were discarded.

The plantation density was 3 × 2 m (3 m between rows and 2 m between plants). The application of fertilizers and irrigation water was supplied by drip fertigation in accordance with the common local conditions for commercial papaya orchards. The applied fertilizers were around 180 (N), 80 (P_2_O_5_), 270 (K_2_O), and 110 (CaO) g/plant, and the requirement of irrigation water was estimated following the recorded data of the agrometeorological station of the location and the application of the evapotranspiration reference. Thus, a total of 35–84 L/plant was applied weekly, and such amounts were distributed with a frequency of three irrigation times a week.

### 2.2. Water Deficit Treatments

The water deficit regimes were initiated when the first fruits reached the stage of harvest (February under our experimental conditions). Based on the recommended irrigation amounts, we established the following treatments: (a) control, plants irrigated at field capacity (100% of the recommended dose of water); (b) moderate deficit irrigation (“MDI”, plants irrigated with 66% of the recommended dose of water); and (c) severe deficit irrigation (“SDI”, plants irrigated with 50% of the recommended dose of water). This irrigation system was applied from February to September in our subtropical conditions.

### 2.3. Plant Growth

To evaluate the effects of water stress on plant growth, functional leaf number per plant, stem height and perimeter were monitored approximately once a month throughout 202 days from February until September in the studied cultivars. In each date only photosynthetically active leaves (functional leaves) were recorded discarding senescent and dried old leaves. Stem height was measured from ground level to apex, and stem perimeter was determined from 0.5 m-height above the ground to maintain the same origin point of measurement.

### 2.4. Fruit Growth, Yield, and Total Soluble Solutes (SST)

The number of collected fruits per plant was determined periodically at each date of harvest (0, 29, 64, 91, 120, 147, 174, and 202 days after treatment (DAT)) according to the established fruit ripening stage (a third part of the fruit peel exhibited an orange-yellow color). Fruit size was determined by measuring the fruit length (cm), and perimeter (cm) of the middle part of the fruit, and individual fruit fresh weight determined at each date of harvest. In addition, the TSS were determined by means of a digital refractometer ATAGO (model PAL-1 Atago Co., Tokyo, Japan). All these variables, excluding fruit number were determined in each date by using three fruits per plant and three plants per treatment (n = 9) randomly distributed only at Day 0 of harvest, the number of fruits used was one fruit per plant and nine plants per treatment (n = 9) due to the low crop yield.

### 2.5. Fruit Mineral Analyses

The concentration of mineral nutrients (N, P, K^+^, Ca^2+^, Mg^2+^, Na^+^, and Cl^−^) was determined in flesh sections from fruits collected during the following harvesting dates: 29, 91, 120, 147, 174 and 202 DAT. For the analysis of N, P, K^+^, Ca^2+^, Mg^2+^, and Na^+^, wet mineralization was performed accordingly to Brayton [22] with little modifications. In brief, 3 g of fruit flesh fresh weight was introduced into Hach 100 mL volumetric flask and 4 mL of H_2_SO_4_ (98%) was added, and once the Hach digestor reached 440 °C the sample was placed on the heater, boiled for 4 min, and subsequently 10 mL H_2_O_2_ (50%) was added keeping the sample boiling for 1 min. Then, the sample was cooled, and the digest diluted as reported in the used procedure. The different mineral contents were analyzed as follows: Nitrogen: once the sample proceeded from the wet mineralization is ready, the nitrogen concentration was determined with the Kjeldahl method using an AutoAnalyzer II Technicon (Technicon Instrument Corporation, Tarrytown, NY, USA) in accordance with Bran and Luebbe [23]; phosphorus: this macronutrient was analyzed according to the methods reported in Technicon [24] and its concentration was measured with colorimetry at 420 nm as the vanadomolybdo-phosphoric acid complex.

Potassium, calcium, magnesium, and sodium: the concentrations of these macro and micro-elements cations were measured by atomic absorption spectrophotometry following the procedures reported in Technicon [24] and Perkin-Elmer [25].

Chloride: To determine the concentration of this anion in the fruits, 2 g of triturated flesh sample was mixed with 25 mL of distilled water and stirred for 10 min. Subsequently, the determination was made using a Metrohm Automatic Titrator (Metrohm AG, Ionenstrasse, Herisau, Switzerland) according to Bruttel and Seifert [26].

### 2.6. Statistical Analyses

For each cultivar, a total of 27 plants distributed in three blocks with nine plants per block and three plants per treatment and block was used. Statistical analyses were performed using Statistix 10 (Tallahassee, FL, USA). An analysis of variance (ANOVA) was applied and data were compared for each cultivar using the least significance difference test (LSD) at *p* < 0.05. This analysis was performed on all variables measured in each date corresponding to plant and fruit growth, and production of each cultivar by comparing, as appropriate, between treatments.

## 3. Results

### 3.1. Plant Growth

In well-watered plants, “Intenzza” and “Siluet” were registered between approx. 46–49 and 42–48 leaves per plant, respectively (Table 1). Water deficit differentially reduced leaf number in both cultivars. Thus, in “Intenzza” water supply regimes [(severe deficit irrigation (“SDI”, 50% watering) or moderate deficit irrigation (“MDI”, 66% watering)] induced a similar reduction of functional leaf number (between 10 and 19%) that initiated 64 DAT and continued throughout the experimental period. However, in “Siluet” the major decrease of leaf number was induced by “SDI” (19–35%) than by “MDI” (8.5–15%) compared to control and took place 35 days earlier than in “Intenzza”. Regarding stem growth, the reduction of its height induced by water stress begun much later in “Intenzza” (174 DAT) with respect to “Siluet” (91 DAT), independently of the stress severity (Table 2). The decline of stem height by drought reached 12 and 25% in comparison with the control at the end of the experiment in moderate- and severe-stressed “Intenzza” plants, respectively (Table 2). In “Siluet”, “SDI” continuously reduced stem length from 91 DAT until the end of the experiment. This variable of growth was 15% and 33% lesser in plants subjected to “MDI” and “SDI”, respectively than in the control at 202 DAT.

The impact of both stress regimes on stem perimeter was similar in the two cultivars, although the reduction was initiated earlier in “Intenzza” (29 DAT) than in “Siluet” (64 DAT). The decrease of this parameter was 45 and 57% compared to control in “Intenzza” and “Siluet” plants, respectively at the end of the experiment (Table 3). Moreover, independently of the applied irrigation system, “Intenzza” exhibited major increase of stem growth (height and perimeter) compared to “Siluet” during the whole experimental period (Table 2 and Table 3).

### 3.2. Fruit Growth and Yield

Fruit growth was assessed through its length (Table 4) and girth (Table 5) along the experimental period. In “Siluet”, “SDI” significantly reduced fruit length from 64 to 202 DAT; however, “MDI” showed similar behavior as control on this parameter. By contrast, in “Intenzza” both stress treatments likewise decreased fruit length during the last three dates of harvest, although the reduction of this variable was initiated 64 and 120 DAT under “SDI” and “MDI”, respectively (Table 4). Regarding fruit width, a parallel decrease was induced in “Intenzza” by water scarcity independently of its extent compared to control since the 91st day of water restriction; however, in “Siluet” only “SDI” decreased fruit girth from the same date until the end of the trial (Table 5).

Concerning fruit yield, the effect of water deficit was assessed by means of the harvested fruit number per plant and date (Table 6), and the average of single fruit fresh weight at harvest (Table 7). In general, the collected fruit number in “Intenzza” was similar between control and both water-stressed plants during the experimental period, except two dates. Accurately, the number of harvested fruits in plants subjected to “MDI” and “SDI” was higher than that of the control 120 and 202 DAT, respectively. Nevertheless, in “Siluet” the behavior of fruit harvest induced by water deficit regime was different than that of “Intenzza”. Thus, a major fruit number was collected in stressed plants at 64 and 120 DAT than in control ones; in contrast, afterwards the number of yielded fruits in control was higher than in plants subjected to both stress treatments 147 and 202 DAT (Table 6). On the other hand, water deficit independently of its intensity continuously decreased fruit fresh weight (FW) since 29 DAT until the end of the experiment in “Intenzza”; however, in “Siluet” the effects of water stress were established later (64 DAT) compared to “Intenzza” (Table 7). Hence, in “Siluet” “SDI” significantly reduced FW since 64 until 202 DAT with respect to control; however, “MDI” only decreased this variable in one date (64 DAT).

### 3.3. Total Soluble Solids Contents in Harvested Fruits

Water supply did not modify the fruit total soluble solids (TSS) levels that oscillated between 10- and 12-degree Brix (°Brix) in fruits of well- or deficit-irrigated “Siluet” plants (Table 8). However, both water stress treatments increased the °Brix of “Intenzza” fruits in several harvest dates (29, 91, 147, 174, and 202 DAT) compared to the control. These values were similar between fruits of plants exposed to “MDI” or “SDI”. It is interesting to note that the measured fruit TSS levels were lower in “Intenzza” than in “Siluet” throughout the experimental period at each date independently of the water regime. In addition, in “Intenzza” a negative correlation occurred between fruit TSS and fruit FW throughout the experimental period irrespective of the watering system, and even these variables are highly correlated under “SDI” treatment. In contrast, no significant correlation between these parameters was reflected in fruits harvested from “Siluet” plants (Table 9). Regarding fruit TSS and fruit water content, a high negative correlation was noticed between both variables along the experimental period, and such correlation was more pronounced especially in fruits of “Intenzza” plants cultivated under “MDI” and “SDI” compared to control. In addition, a great negative correlation was found between these parameters particularly under “SDI” in “Siluet” plants (Table 9).

### 3.4. Fruit Mineral Nutrient Contents

Fruit N concentrations varied approximately between 75.6 and 133.2 mg/100 g FW in control and treated plants of both cultivars (Table 10). The applied deficit irrigation system did not alter the N content, and only a slight decrease in control fruits compared to deficit-watered ones was observed at the end of the experiment in “Siluet”. Fruit P levels varied between 10.0 and 43.5 mg/100 g FW during the experimental period in well- or deficit irrigated “Intenzza” and “Siluet” plants (Table 10). Such concentrations were eventually altered 91 and 174 days after the establishment of the water irrigation regime in comparison to control in “Intenzza”. Thus, at 91 DAT fruits of plants subjected to “SDI” accumulated more P than control and “MDI” ones, and at 174 DAT a major concentration of P was measured in fruits of “MDI” plants (Table 10). In “Siluet” fruits, a reduction of P content in water-stressed (“SDI” and “MDI”) plants was determined 29 DAT compared to control, and thereafter a small decrease of this element in fruits of “SDI” plants with respect to control was found 174 DAT.

The levels of K^+^ oscillated between 256.5 and 394.9 mg/100 g FW during the harvest periods in both cultivars and under the different amounts of water supply. Water shortage did not modify K^+^ concentration except in one date (120 DAT) throughout the experimental period where fruits of “SDI” plants contained more K^+^ (24%) than control in “Intenzza” (Table 10).

Calcium concentrations in fruits of the studied cultivars varied between 3.0 and 13.4 mg/100 g FW during the periods of harvest, and no changes were observed between watering treatments (Table 10).

Magnesium content oscillated between approx. 16 and 31.5 mg/100 g FW in control and deficit-irrigated plants throughout the experimental period (Table 10). Watering restriction did not alter the concentrations of this element in both cultivars.

Sodium concentrations varied between 14.5–30.3 and 24.8–44.6 mg/100 g FW in all the treatments at the different dates of harvest in “Intenzza” and “Siluet”, respectively (Table 11). Water scarcity did not modify the concentration of this element in both cultivars. However, fruits of “Siluet” accumulated more Na^+^ than those of “Intenzza” during various dates of harvest in control as well as in water-stressed plants. In addition, Cl^−^ content was the highest salt ion content compared to Na^+^ in the fruits of control and deficit-irrigated plants. Again, water shortage did not alter the content of these ions with respect to control, except for one date (91 DAT). In such a case, “SDI” decreased Cl^−^ in “Intenzza” fruits compared to “CT” and “MDI”, whereas in “Siluet” both water stress treatments increased Cl^−^ levels with respect to “CT”.

## 4. Discussion

### 4.1. Plant Growth

Pant growth was expressed as functional leaf number, stem height, and perimeter throughout the period of irrigation management. Water deficit differentially decreased leaf number in “Intenzza” and “Siluet” (Table 1). In “Intenzza” both water stress regimes (“MDI” and “SDI”) induced a similar reduction of functional leaf number that initiated 64 DAT; however, in “Siluet” the effects of “MDI” on leaf abscission was established later (120 DAT) and the decrease of leaf number was induced by “SDI” that took place since 29 DAT. These results may indicate that papaya faces water deficit by removing part of its aged leaves and such responses depend on genotype and stress intensity. In general, the senescence of the old leaves incited by drought could decrease the levels of the photosynthetic pigments leading to the depletion of photosynthetic capacity of the plants [14,15]. This is consistent with our previous experiments in papaya seedlings subjected to progressive drought [6,27]. In this context, water stress decreases the functional leaf number by an induction of the senescence of the oldest leaves firstly and their abscission subsequently, and a minor new leaf emission per plant. It has also been shown that leaf abscission increases parallelly to the rise of the foliar and radicular abscisic acid and to the decrease of the photosynthetic rate, stomatal conductance, and transpiration rate [6,27]. In general, plants respond to water deficit through various strategies, such as growth pattern dynamic, leaf rolling, root and shoot traits, accumulation of compatible solutes and osmotic adjustment, transpiration efficiency, and hormonal regulation [28,29,30]. Regarding stem growth, water deficit independently of its intensity delayed the decrease of stem height in “Intenzza” with respect to “Siluet”. At the end of the trial, the decline in stem height by drought was considerable especially under “SDI” (Table 2). Concerning stem circumference, water shortage showed similar pattern of growth decrease induced earlier in “Intenzza” than in “Siluet”, and such reduction was relatively more pronounced in “Siluet” at the end of the experiment (Table 3). In the same way these variables of growth are affected by drought under different intensities in apple trees [19]. Collectively, in terms of plant growth the effects of water deficit (“MDI” or “SDI”) seem to be relatively less harmful in “Intenzza” than in “Siluet”. This dissimilar response to water restriction could be associated to several factors, such as vigor, since “Intenzza” is more vigorous than “Siluet”, genotypic variability, cultivar features related to osmotic adjustments, and other specific adaptive responses to abiotic stress. In our previous studies we reported that papaya plants decrease the leaf number and growth such as leaf length, fresh and dry weight (DW), and relative growth rate expressed as stem DW under water deficit regimes [6,27,31]. It has also been reported in earlier works that soil water potential restriction negatively affects plant growth and development of papaya through the reduction of plant size, flowering, and fruiting [32]. Moreover, even under short-term drought period, papaya seedlings experience a decrease of stem height and diameter, canopy width, and leaf area [33].

### 4.2. Fruit Growth and Yield

The applied regulated deficit irrigation altered the normal fruit growth measured periodically as length (Table 4) and width (Table 5). Thus, in “Siluet” only “SDI” declined both variables and no impact on growth was produced by “MDI”. Nevertheless, in “Intenzza”, “MDI” as well “SDI” similarly decreased fruit growth during several dates of harvest. Regarding the crop yield, water deficit treatments did not reduce fruit number (Table 6) and even the number of collected fruits was higher than control in two dates (120 and 202 DAT) in “Intenzza”. In “Siluet”, the imposed irrigation system affected the amounts of fruits harvested during several dates by increasing the quantity of mature fruits in stressed plants (64 and 120 DAT), and later a major number of fruits was collected from control ones (147 and 202 DAT). On the other hand, it is important to note that water deficit had a remarkable negative effect on FW independently of its intensity in “Intenzza”; however, in “Siluet” the onset of FW decrease was induced over one month later than in “Intenzza” and exclusively by “SDI” (Table 7). In “Siluet”, “MDI” only decreased this variable in one date indicating a lower impact of this treatment in fruit growth compared to “SDI” (Table 7); in addition, fruit enlargement phases, size and yield decrease under drought conditions in papaya [32]. In other species, such as grapevine, water deficit decreases the cluster and berry size, the number of berries, and yield [20], and in tomato plants it reduces yield and dry matter production [21].

These results presented here may suggest that “Siluet” could be irrigated through “MDI” treatment without noticeable negative effects on its fruit growth. Moreover, it should be mentioned that the major fruit number was collected during June (120 DAT) and July (147 DAT) in both cultivars, and water deficit clearly accelerated fruit harvest by prompting a greater production in June (one month earlier compared to control plants). In grapevines, water stress also accelerates fruit ripening [20].

Concerning fruit growth and biomass, our results showed similar trends (at least in plants subjected to “MDI”) as those reported in *Carica papaya* “Sinta” subjected to drought during three months without water supply [34]. In that study drought decreases fruit weight, length, width, flesh thickness, and seed weight. In addition, in an experimental system using two genotypes differing in leaf chlorophyll content, “Golden” (pale-green leaf) and “Aliança” (dark-green leaf), it has been reported a dissimilar response to soil water stress between both genotypes [35]. Thereby, water deficit does not alter the “Golden” fruit number while it reduces this parameter by 83% in “Aliança” in comparison with irrigated plants, suggesting that pale-green leaf papaya genotypes could cope with water stress [35].

### 4.3. Total Soluble Solids Content in Harvested Fruits

The total soluble solids are critical indicators of fruit organoleptic quality in papaya and a prerequisite for the commercialization of this tropical fruit. Data presented here determined under subtropical conditions exhibited values of TSS (10–12 °Brix) relatively lower than those reported by the manufacturer *Semillas del Caribe* (semillasdelcaribe.com.mx, accessed on 29 September 2022) in tropical climates (“Intenzza”, 10–13 °Brix and “Siluet”, 13–15 °Brix). The established deficit irrigation system (“MDI” or “SDI”) enhanced fruit TSS values during almost the whole harvest periods in “Intenzza”; however, it did not modify these parameters in “Siluet” along the trial. Furthermore, the measured fruit TSS levels were lower in “Intenzza” than in “Siluet” throughout the experimental period at each date independently of the water regime. These results indicated that the variation in sugar accumulation in well- or deficit-irrigated papaya plants may depend on cultivar/genotype, and these findings coincide with those reported in other species [36]. It is interesting to note that both deficit-irrigation systems induced an antagonistic change between fruit FW and TSS in “Intenzza” plants. Thus, these treatments provoked a decrease of FW and an increase of °Brix in the fruits, and consequently a high negative correlation occurred between both variables especially under “SDI” treatment in “Intenzza” (Table 9). Likewise, fruit TSS levels highly correlated with fruit water content in fruits of “Intenzza” plants subjected to “MDI” and “SDI” and particularly under “SDI” in “Siluet” plants (Table 9). The increase of TSS and soluble sugars under drought conditions has been reported in several species, such as papaya [34], apple [19,37], pear [38]), tomato [21,39,40], and strawberry [41]. In strawberry the changes in the major sugars and organic acids in leaves and fruits could be linked to an osmotic adjustment approach of the plants to alleviate the effects of drought [41]). Being the TSS parameter a key factor for papaya commercialization, it is plausible the finding that the relatively loss of FW is accompanied by an increase in sugar content and finally by an improvement of fruit quality. According to its agronomic characteristics under tropical and subtropical conditions, “Intenzza” canopy is more vigorous, and its fruit size is higher than that of “Siluet”; however, values of fruit TSS are lower in “Intenzza” than in “Siluet”. Therefore, an adequate water regime management may improve fruit quality of this deficient cultivar in TSS for market exigency at least in subtropical regions.

### 4.4. Fruit Mineral Nutrient Contents

In general, the mineral elements deficiency negatively affects plant metabolism and subsequently fruit growth and development, yield, nutrient content, and quality of fruits in several plant species [42]. It has been reported that papaya fruit is a useful source of various mineral elements, especially K^+^, Mg^2+^, and B [43]. In previous experiments using papaya seedlings we show that drought conditions do not change the nitrogen concentration and even increase K^+^, Na^+^, and Cl^−^ levels in leaf and root organs [6]. Regarding N, the same trend was observed here throughout the experimental period except for a minor reduction in fruits of control with respect to those of stressed plants at the end of the trial in “Siluet” (Table 10). The content of P in “Intenzza” fruits was similar or higher in water-stressed plants than in control; however, in “Siluet” only a modest decrease of this element was measured in dehydrated plants compared to regularly watered ones in few dates of harvest. In other experimental systems, the levels of P in papaya fruits show values within the same intervals as our findings (approx. 12.5 mg/100 g FW [44]) or much lower concentrations (about 5.4 mg/100 g FW [45], and between 5 and 6.9 mg/100 g FW [46]). Water deficiency does not decrease K^+^ levels in the studied cultivars and even increased its content in one date (120 DAT) in fruits of “Intenzza” plants subjected to “SDI” compared to control (Table 10). Generally, the levels of this macronutrient determined here were significantly higher than those observed in other regions worldwide [41,42,43,44,45,46]. Several factors should be involved in these dissimilar concentrations which we can mention among others, the genotypes, the amounts of fertilizers supplied, the soil and water characteristics, the climate, etc. Concerning Ca^2+^ concentrations, watering treatments did not induce changes in both cultivars compared to control (Table 10). In addition, unlike K^+^ and P, the concentrations of Ca^2+^ obtained in other studies were similar or higher than the results determined here. For instance, values of 24.9, 11.1, 14.7, and 19.6–23.9 mg/100 g FW have been measured in fruits of different genotypes [42,44,45,47], respectively. Water constraint system does not disturb the concentrations of Mg^2+^ in both cultivars. In some experiments [44,46], the levels of Mg^2+^ coincide within the interval of our data, although others studies indicate lower concentrations [45,47].

It is important to note that the range of Na^+^ contents was lower in “Intenzza” than in “Siluet” during various dates of harvest in all the applied watering systems (Table 11). Nevertheless, water deficit did not affect the concentration of this element in each cultivar. It is interesting to mention that these levels are considerably higher than those previously found [44,45]. The experiments of Tripathi et al. [46] show concentrations of Na^+^ much lower (2.7 and 3.2 mg/100 FW) than those obtained here. Such large differences in Na^+^ accumulation may be associated to the irrigation water quality and soil chemical composition. In addition, Cl^−^ was the highest salt ion content compared to Na^+^ in the fruits of control and deficit-irrigated plants. Again, water shortage did not alter the content of these ions in the fruits with respect to control, although in our previous works drought increases the foliar level of this element in papaya seedlings [6].

In summary, the most abundant macronutrient in papaya fruits was K^+^ followed by N, Mg^2+^, P, and Ca^2+^ independently of the genotype and the applied water regimes (Table 10). These findings indicate that water deficit did not notably impair the mineral composition of the fruit and maintained a suitable organoleptic quality leastwise at nutritional level. This achievement was obtained through the applied deficit irrigation strategy by reducing water supply and maintaining the fertilizer amounts as the control level.

## 5. Conclusions

Taken together, data might suggest that despite the relatively negative effects induced by the regulated water deficit on plant and fruit growth, the imposed treatments did not considerably impair the concentrations of the most mineral elements in the fruits of both cultivars and improved the TSS levels in “Intenzza”. In addition, at least “Siluet” could be cultivated successfully under “MDI” regime without remarkable damages on fruit production and consequently saving 33% of water supply. Finally, further research is required to provide additional insights into the management of deficit irrigation and fertilization to improve the water and nutrient use efficiencies with the aim to obtain a better fruit yield and organoleptic quality of different papaya genotypes.

## Figures and Tables

**Table 1:** Functional leaf number in control (CT, 100% watered), moderate deficit irrigated (MDI, 66% watered) and severe deficit irrigated (SDI, 50% watered) plants of “Intenzza” and “Siluet”. For each cultivar, data are means ± SE and each value was determined in three plants with three replicates per treatment (n = 9). Data were compared for each date using the least significant difference (LSD) test. Values in each line and date followed by a distinct letter differ significantly at *p* < 0.05 in each cultivar. DAT = days after treatment.

Functional Leaf Number						
	Intenzza			Siluet		
DAT	CT	MDI	SDI	CT	MDI	SDI
0	49.4 ± 1.2a	44.6 ± 1.1a	47.5 ± 0.9a	48.3 ± 0.8a	45.8 ± 0.7a	43.5 ± 0.6a
29	46.5 ± 1.3a	42.3 ± 1.1a	43.8 ± 1.0a	45.1 ± 0.9a	42.3 ± 0.4a	36.6 ± 0.4b
64	45.8 ± 1.4b	41.2 ± 1.2a	41.5 ± 1.0a	43.1 ± 1.1a	39.2 ± 0.4a	27.7 ± 0.6b
91	48.4 ± 1.6b	42.8 ± 1.2a	41.9 ± 1.0a	42.9 ± 1.5a	38.2 ± 0.5ab	28.3 ± 0.4bc
120	48.3 ± 1.5b	42.0 ± 1.3a	41.5 ± 0.9a	42.3 ± 1.4a	36.9 ± 0.4b	31.7 ± 0.3c
147	47.3 ± 1.5b	40.6 ± 1.1a	38.3 ± 1.1a	42.7 ± 1.0a	36.2 ± 0.4b	30.9 ± 0.3c
174	47.7 ± 1.4b	39.2 ± 1.2a	38.7 ± 1.2a	42.4 ± 1.4a	36.3 ± 0.6b	33.3 ± 0.4b
202	47.3 ± 2.8b	41.2 ± 1.0a	41.5 ± 0.9a	46.4 ± 1.2a	41.1 ± 0.5b	36.6 ± 0.5c

**Table 2:** Increased stem height in control (CT, 100% watered), moderate deficit irrigated (MDI, 66% watered) and severe deficit irrigated (SDI, 50% watered) plants of “Intenzza” and “Siluet”. For each cultivar, data are means ± SE and each value was determined in three plants with three replicates per treatment (n = 9). Data were compared for each date using the least significant difference (LSD) test. Values in each line and date followed by a distinct letter differ significantly at *p* < 0.05 in each cultivar. DAT = days after treatment.

Increased Stem Height (%)						
	Intenzza			Siluet		
DAT	CT	MDI	SDI	CT	MDI	SDI
0	1.3 ± 0.3a	1.5 ± 0.0a	1.0 ± 0.1a	0.1 ± 0.0a	0.1 ± 0.0a	0.1 ± 0.0a
29	1.1 ± 0.2a	1.6 ± 0.1a	1.0 ± 0.1a	0.4 ± 0.2a	0.2 ± 0.1a	0.2 ± 0.1a
64	1.7 ± 0.2a	2.1 ± 0.1a	1.5 ± 0.1a	0.5 ± 0.1a	0.5 ± 0.1a	0.3 ± 0.1a
91	2.7 ± 0.3a	2.8 ± 0.1a	2.3 ± 0.1a	1.3 ± 0.3a	0.9 ± 0.1ab	0.5 ± 0.1b
120	5.2 ± 0.3a	4.8 ± 0.3a	4.4 ± 0.1a	3.7 ± 0.3a	2.9 ± 0.3b	2.6 ± 0.1b
147	8.1 ± 0.2a	7.7 ± 0.2a	7.3 ± 0.2a	5.9 ± 0.1a	5.7 ± 0.2ab	5.3 ± 0.2b
174	11.8 ± 0.3a	10.3 ± 0.3b	10.2 ± 0.2b	9.9 ± 0.3a	8.6 ± 0.3b	7.1 ± 0.2c
202	14.5 ± 0.4a	12.7 ± 0.4b	10.8 ± 0.4c	13.1 ± 0.4a	11.1 ± 0.4b	8.8 ± 0.4c

**Table 3:** Increased stem perimeter in control (CT, 100% watered), moderate deficit irrigated (MDI, 66% watered) and severe deficit irrigated (SDI, 50% watered) plants of “Intenzza” and “Siluet”. For each cultivar, data are means ± SE and each value was determined in three plants with three replicates per treatment (n = 9). Data were compared for each date using the least significant difference (LSD) test. Values in each line and date followed by a distinct letter differ significantly at *p* < 0.05 in each cultivar. DAT = days after treatment.

Increased Stem Perimeter (%)						
	Intenzza			Siluet		
DAT	CT	MDI	SDI	CT	MDI	SDI
0	5.9 ± 0.3a	5.5 ± 0.3a	4. 6 ± 0.3a	2.6 ± 0.3a	2.1 ± 0.2a	2.5 ± 0.2a
29	8.2 ± 0.6a	6.3 ± 0.3b	5.1 ± 0.4b	4.2 ± 0.6a	2.5 ± 0.2a	2. 5 ± 0.3a
64	11.6 ± 0.7a	8.1 ± 0.3b	6.8 ± 0.4b	7.4 ± 0.7a	3.8 ± 0.3b	4.2 ± 0.4b
91	13.4 ± 0.8a	9.1 ± 0.3b	7.2 ± 0.5b	8.8 ± 0.8a	4.3 ± 0.3b	4.6 ± 0.5b
120	14.7 ± 0.9a	9.6 ± 0.4b	7.5 ± 0.4b	9.5 ± 0.9a	4.8 ± 0.4b	4.4 ± 0.4b
147	16.1 ± 0.1a	10.4 ± 0.4b	8.3 ± 0.5b	10.2 ± 1.0a	5.3 ± 0.4b	4.6 ± 0.5b
174	18.2 ± 1.0a	11.5 ± 0.5b	9.6 ± 0.5b	11.7 ± 1.0a	6.1 ± 0.5b	5.0 ± 0.5b
202	19.5 ± 1.1a	12.0 ± 0.5b	10.6 ± 0.6b	12.5 ± 1.1a	7.2 ± 0.5b	5.3 ± 0.6b

**Table 4:** Fruit length in control (CT, 100% watered), moderate deficit irrigated (MDI, 66% watered) and severe deficit irrigated (SDI, 50% watered) plants of “Intenzza” and “Siluet”. For each cultivar, data are means ± SE and each value was determined by using three fruits per plant and three plants per treatment (n = 9). Data were compared for each date using the least significant difference (LSD) test. Values in each line and date followed by a distinct letter differ significantly at *p* < 0.05 in each cultivar. DAT = days after treatment.

Fruit Length (cm)						
	Intenzza			Siluet		
DAT	CT	MDI	SDI	CT	MDI	SDI
0	12.1 ± 0.1a	11.9 ± 0.2a	12.5 ± 0.1a	10.6 ± 0.1a	10.9 ± 0.2a	10.6 ± 0.2a
29	13.8 ± 0.1a	14.6 ± 0.2a	13.5 ± 0.2a	11.6 ± 0.1a	12.4 ± 0.2a	11.8 ± 0.2a
64	17.3 ± 0.2a	17.0 ± 0.2a	15.7 ± 0.2b	14.4 ± 0.2a	14.2 ± 0.2a	13.5 ± 0.2b
91	19. 5 ± 0.2a	17.5 ± 0.2a	18.7 ± 0.2b	16.0 ± 0.2a	15.5 ± 0.4a	14.7 ± 0.2b
120	20.3 ± 0.2a	18.2 ± 0.2b	19.3 ± 0.2c	16.5 ± 0.2a	16.3 ± 0.2a	15.1 ± 0.2b
147	21.0 ± 0.2a	19.0 ± 0.2b	19.7 ± 0.2b	17.0 ± 0.2a	17.0 ± 0.2a	15.6 ± 0.2b
174	21.4 ± 0.3a	18.7 ± 0.3b	19.5 ± 0.3b	16.9 ± 0.3a	16.9 ± 0.3a	15.7 ± 0.3b
202	21.2 ± 0.3a	18.5 ± 0.3b	19.6 ± 0.2b	16.9 ± 0.3a	16.9 ± 0.4a	15.7 ± 0.3b

**Table 5:** Increased fruit girth in control (CT, 100% watered), moderate deficit irrigated (MDI, 66% watered) and deficit irrigated (SDI, 50% watered) plants of “Intenzza” and “Siluet”. For each cultivar, data are means ± SE and each value was determined by using three fruits per plant and three plants per treatment (n = 9). Data were compared for each date using the least significant difference (LSD) test. Values in each line and date followed by a distinct letter differ significantly at *p* < 0.05 in each cultivar. DAT = days after treatment.

Increased Fruit Girth (%)						
	Intenzza			Siluet		
DAT	CT	MDI	SDI	CT	MDI	SDI
0	17.4 ± 0.3a	17.6 ± 0.3a	18.8 ± 0.2a	17.4 ± 0.3a	18.5 ± 0.3a	18.2 ± 0.2a
29	20.9 ± 0.3a	20.4 ± 0.3a	21.4 ± 0.2a	19.8 ± 0.3a	20.8 ± 0.3a	19.8 ± 0.2a
64	25.7 ± 0.4a	24.0 ± 0.3a	24.9 ± 0.3a	23.5 ± 0.4a	23.9 ± 0.3a	22.8 ± 0.3a
91	29.0 ± 0.4a	26.5 ± 0.3b	27.2 ± 0.3b	26.0 ± 0.4a	26.1 ± 0.3a	24.8 ± 0.3b
120	30.2 ± 0.4a	27.5 ± 0.3b	28.2 ± 0.3b	27.1 ± 0.4a	27.0 ± 0.3a	25.3 ± 0.3b
147	31.4 ± 0.5a	27.9 ± 0.3b	28.2 ± 0.3b	28.2 ± 0.4a	27.9 ± 0.3a	26.4 ± 0.2b
174	30.8 ± 0.5a	27.2 ± 0.4b	28.9 ± 0.3b	29.1 ± 0.5a	27.9 ± 0.4a	26.7 ± 0.3b
202	30.9 ± 0.2a	27.3 ± 0.5b	28.4 ± 0.2b	29.3 ± 0.2a	28.1 ± 0.1a	26.8 ± 0.3b

**Table 6:** Harvested fruit number per plant in control (CT, 100% watered), moderate deficit irrigated (MDI, 66% watered), and severe deficit irrigated (SDI, 50% watered) plants of “Intenzza” and “Siluet”. For each cultivar, data are means ± SE and each value was determined in three plants with three replicates per treatment (n = 9). Data were compared for each date using the least significant difference (LSD) test. Values in each line and date followed by a distinct letter differ significantly at *p* < 0.05 in each cultivar. DAT = days after treatment.

Fruit Number						
	Intenzza			Siluet		
DAT	CT	MDI	SDI	CT	MDI	SDI
0	1.3 ± 0.3a	2.5 ± 0.5a	2.5 ± 0.4a	0.9 ± 0.4a	1.0 ± 0.5a	1.7 ± 0.5a
29	3.3 ± 0.4a	4.3 ± 0.7a	4.4 ± 0.5a	4.7 ± 0.6ab	5.1 ± 0.43a	3.7 ± 0.5b
64	2.5 ± 0.5a	4.1 ± 0.9a	4. 9 ± 0.9a	3.3 ± 0.5a	5.7 ± 0.63b	6.2 ± 0.7b
91	7.6 ± 1.4a	10.1 ± 1.4a	9.8 ± 1.4a	9.1 ± 1.3a	11.5 ± 1.5a	11.0 ± 0.9a
120	15.1 ± 1.7a	21.2 ± 2.0b	18.6 ± 1.4ab	16.1 ± 1.1a	21.5 ± 1.5b	22.1 ± 1.2b
147	15.4 ± 1.6a	11.8 ± 1.2a	15.1 ± 1.3a	22.3 ± 1.84a	16.9 ± 1.3b	16.7 ± 1.5b
174	7.8 ± 1.1a	8.1 ± 0.8a	7.7 ± 0.9a	13.5 ± 1.0a	13.2 ± 0.9a	13.4 ± 1.2a
202	2.3 ± 0.8a	2.0 ± 0.6	3.8 ± 0.7b	8.9 ± 1.01a	5.4 ± 0.7b	6.8 ± 0.7ab

**Table 7:** Fruit fresh weight in control (CT, 100% watered), moderate deficit irrigated (MDI, 66% watered) and severe deficit irrigated (SDI, 50% watered) plants of “Intenzza” and “Siluet”. For each cultivar, data are means ± SE and each value was determined by using three fruits per plant and three plants per treatment (n = 9). Data were compared for each date using the least significant difference (LSD) test. Values in each line and date followed by a distinct letter differ significantly at *p* < 0.05 in each cultivar. DAT = days after treatment.

Fruit Fresh Weight (g)						
	Intenzza			Siluet		
DAT	CT	MDI	SDI	CT	MDI	SDI
0	1759.9 ± 97.2a	1660.9 ± 99.5a	1692.6 ± 97.8a	1085.5 ± 91.4a	986.1 ± 12.2a	1041.6 ± 35.4a
29	1854.4 ± 70.4a	1599.4 ± 51.3b	1592.8 ± 30.3b	1048.2 ± 85.4ab	1064.9 ± 10.2a	949.3 ± 33.2b
64	1584.2 ± 61.3a	1330.7 ± 49.5b	1282.8 ± 28.5b	1048.2 ± 30.8a	905.8 ± 11.2b	816.7 ± 16.3c
91	1307.8 ± 37.2a	1081.5 ± 25.4b	1095.0 ± 24.6b	764.7 ± 28.1a	781.1 ± 5.2a	663.2 ± 10.2b
120	1144.6 ± 23.5a	978.6 ± 15.5b	892.9 ± 18.4b	715.3 ± 25.4a	711.6 ± 6.4a	625.1 ± 8.8b
147	1045.1 ± 17.3a	869.2 ± 16.2b	807.3 ± 12.5b	681.5 ± 18.4a	669.4 ± 6.5a	583.2 ± 14.2b
174	842.7 ± 22.7a	689.4 ± 16.9b	632.8 ± 14.3b	548.9 ± 16.7a	518.5 ± 4.4a	438.8 ± 10.3b
202	533.2 ± 35.5a	439.8 ± 24.1b	420.0 ± 22.3b	339.2 ± 15.3a	299.1 ± 5.6a	272.1 ± 10.8b

**Table 8:** Fruit total soluble solids in control (CT, 100% watered), moderate deficit irrigated (MDI, 66% watered), and severe deficit irrigated (SDI, 50% watered) plants of “Intenzza” and “Siluet”. For each cultivar, data are means ± SE and each value was determined by using three fruits per plant and three plants per treatment (n = 9). Data were compared for each date using the least significant difference (LSD) test. Values in each line and date followed by a distinct letter differ significantly at *p* < 0.05 in each cultivar. DAT = days after treatment.

Fruit Total Soluble Solids (°Brix)						
	Intenzza			Siluet		
DAT	CT	MDI	SDI	CT	MDI	SDI
0	9.6 ± 0.3a	10.1 ± 0.2a	9.4 ± 0.2a	12.2 ± 0.2a	11.7 ± 0.6a	11.4 ± 0.2a
29	8.7 ± 0.1a	9.1 ± 0.1b	9.4 ± 0.1c	11.6 ± 0.1a	11.4 ± 0.1a	11.9 ± 0.1a
64	9.7 ± 0.1a	9.7 ± 0.1a	9.4 ± 0.1a	10.4 ± 0.1a	11.1 ± 0.2a	10.7 ± 0.1a
91	8.9 ± 0.2a	9.4 ± 0.1b	9.3 ± 0.1b	10.7 ± 0.1a	10.7 ± 0.1a	10.3 ± 0.2a
120	9.6 ± 0.1a	9.7 ± 0.1a	9.8 ± 0.1a	10.8 ± 0.1a	10.6 ± 0.0a	10.4 ± 0.1a
147	9.7 ± 0.1a	10.5 ± 0.1b	10.4 ± 0.1b	11.2 ± 0.1a	10.9 ± 0.2a	10.8 ± 0.1a
174	10.1 ± 0.1a	10.9 ± 0.1b	11.1 ± 0.1b	11.6 ± 0.2a	11.8 ± 0.2a	12.1 ± 0.1a
202	10.3 ± 0.1a	11.1 ± 0.2b	11.3 ± 0.2b	12.3 ± 0.2a	12.1 ± 0.2a	12.3 ± 0.1a

**Table 9:** Correlation between fruit total soluble solids (TSS) and fruit fresh weight (FW), and fruit water content (WC) in control (CT, 100% watered), moderate deficit irrigated (MDI, 66% watered) and severe deficit irrigated (SDI, 50% watered) plants of “Intenzza” and “Siluet” during the experimental period (202 days). N = 9. *: *p* < 0.05; **: *p* < 0.01; ***: *p* < 0.005. FW (g). Water content (%). TSS (°Brix).

		Intenzza			Siluet	
	CT	MDI	SDI	CT	MDI	SDI
FW vs TSS						
- R^2^	0.516	0.539	0.745	0.061	0.119	0.067
- *p*	0.045	0.038	0.006	0.554	0.402	0.535
- Significance	*	*	**	n.s.	n.s.	n.s.
						
WC vs TSS						
- R^2^	0.701	0.874	0.807	0.537	0.645	0.803
- *p*	0.009	0.001	0.002	0.039	0.016	0.003
- Significance	**	***	***	*	*	***

**Table 10:** Fruit macronutrients contents in control (CT, 100% watered), moderate deficit irrigated (MDI, 66% watered), and severe deficit irrigated (SDI, 50% deficit irrigated) plants of “Intenzza” and “Siluet”. For each cultivar, data are means ± SE and each value was determined by using three fruits per plant and three plants per treatment (n = 9). Data were compared for each date using the least significant difference (LSD) test. Values in each line and date followed by a distinct letter differ significantly at *p* < 0.05 in each cultivar.

Macronutrients (mg/100 g FW)						
	Intenzza			Siluet		
DAT	CT	MDI	SDI	CT	MDI	SDI
**N**						
29	75.6 ± 2.7a	86.2 ± 8.1a	84.2 ± 6.6a	95.7 ± 5.6a	84.1 ± 12.0a	93.9 ± 6.0a
91	100.3 ± 8.4a	98.6 ± 0.9a	105.2 ± 8.1a	108.21 ± 4.3a	85.0 ± 2.6a	109.2 ± 15.7a
120	96.1 ± 5.4a	110.4 ± 1.7a	112.7 ± 6.7a	105.9 ± 6.7a	106.5 ± 1.8a	125.0 ± 6.1a
147	120.6 ± 6.5a	126.0 ± 7.3a	133.2 ± 4.7a	129.0 ± 8.7a	126.4 ± 15.0a	131.9 ± 26.9a
174	93.4 ± 7.5a	120.7 ± 4.3a	108.9 ± 5.5a	114.2 ± 10.9a	116.3 ± 0.4a	128.8 ± 5.0a
202	81.5 ± 11.4a	113.9 ± 3.6a	117.6 ± 15.8a	80.6 ± 2.7a	105.5 ± 1.9b	102.0 ± 9.1b
**P**						
29	25.9 ± 9.2a	30.6 ± 8.3a	14.8 ± 0.1a	40.3 ± 7.7a	16.9 ± 3.6b	15.8 ± 1.4b
91	11.4 ± 1.0ab	10.1 ± 0.1b	15.4 ± 1.5a	10.2 ± 2.5a	10.0 ± 1.3a	15.6 ± 1.0a
120	22.7 ± 8.5a	43.2 ± 9.3a	43.5 ± 9.7a	25.8 ± 5.9a	31.7 ± 3.4a	35.9 ± 4.6a
147	15.6 ± 2.0a	17.6 ± 1.7a	17.6 ± 1.7a	14.7 ± 1.4a	15.4 ± 2.3a	15.5 ± 2.2a
174	13.9 ± 0.2a	18.6 ± 1.1b	11.8 ± 0.6a	14.9 ± 1.7ab	17.6 ± 0.5a	11.5 ± 0.7b
202	17.1 ± 1.5a	21.8 ± 4.3a	17.4 ± 3.3a	12.1 ± 0.8a	13.3 ± 0.7a	14.5 ± 1.8a
**K^+^**						
29	319.3 ± 21.5a	317.3 ± 22.7a	341.5 ± 10.9a	343.6 ± 21.3a	302.9 ± 32.1a	335.6 ± 11.6a
91	345.9 ± 38.1a	307.0 ± 19.3a	320.2 ± 6.1a	344.5 ± 19.5a	316.4 ± 5.2a	389.3 ± 67.1a
120	256.5 ± 27.1b	297.9 ± 7.8ab	337.8 ± 9.9a	288.1 ± 26.1a	279.5 ± 9.9a	349.7 ± 21.0a
147	294.6 ± 9.9a	309.1 ± 8.7a	309.1 ± 8.7a	318.0 ± 55.7a	301.6 ± 14.5a	345.0 ± 28.6a
174	333. 9 ± 18.3a	394.9 ± 26.1a	359.4 ± 18.9a	320.2 ± 14.8a	378.2 ± 30.4a	379.5 ± 13.2a
202	291.6 ± 35.6a	349.8 ± 33.8a	302.4 ± 18.7a	243.3 ± 25.8a	321.5 ± 17.2a	285.6 ± 22.5a
**Ca^2+^**						
29	5.9 ± 0.2a	6.2 ± 0.7a	6.3 ± 0.6a	8.3 ± 2.4a	9.1 ± 0.2a	10.1 ± 3.6a
91	6.6 ± 0.7a	10.2 ± 1.4a	6.2 ± 0.8a	6.3 ± 1.5a	4.8 ± 2.4a	9.1 ± 1.8a
120	3.0 ± 0.4a	6.3 ± 2.4a	4.3 ± 1.0a	3.9 ± 0.4a	4.9 ± 0.6a	3.3 ± 0.9a
147	6.7 ± 0.7a	7.4 ± 1.6a	7.4 ± 1.6a	6.0 ± 0.8a	6.8 ± 1.4a	5.6 ± 0.0a
174	8.1 ± 1.4a	7.9 ± 0.8a	10.7 ± 0.6a	9.1 ± 0.8a	10.1 ± 1.1a	6.5 ± 0.5a
202	6.6 ± 0.6a	9.3 ± 2.1a	13.4 ± 3.7a	10.7 ± 2.3a	10.7 ± 2.6a	8.5 ± 0.9a
**Mg^2+^**						
29	18.5 ± 1.3a	17.6 ± 1.7a	18.8 ± 0.5a	22.0 ± 3.0a	23.0 ± 2.5a	22.5 ± 5.5a
91	18.2 ± 3.4a	21.4 ± 2.2a	16.4 ± 2.2a	17.8 ± 1.5a	16.4 ± 2.7a	21.0 ± 2.3a
120	16.0 ± 0.3a	23.6 ± 3.2a	18.7 ± 1.4a	20.1 ± 1.4a	22.5 ± 1.7a	18.3 ± 1.9a
147	17.6 ± 0.4a	17.6 ± 0.9a	17.6 ± 0.8a	19.7 ± 1.6a	20.8 ± 1.6a	19.4 ± 1.9a
174	17.8 ± 0.5a	21.1 ± 1.3a	23.7 ± 0.9a	23.6 ± 1.4a	25.3 ± 1.9a	21.5 ± 2.3a
202	22.4 ± 1.7a	25.5 ± 4.5a	30.2 ± 7.2a	26.4 ± 1.7a	31.5 ± 4.5a	26.7 ± 0.4a

**Table 11:** Fruit micronutrients contents in control (CT, 100% watered), moderate deficit irrigated (MDI, 66% watered) and severe deficit irrigated (SDI, 50% deficit irrigated) plants of “Intenzza” and “Siluet”. For each cultivar, data are means ± SE and each value was determined by using three fruits per plant and three plants per treatment (n = 9). Data were compared for each date using the least significant difference (LSD) test. Values in each line and date followed by a distinct letter differ significantly at *p* < 0.05 in each cultivar. DAT = days after treatment.

Micronutrients (mg/100 g FW)						
	Intenzza			Siluet		
DAT	CT	MDI	SDI	CT	MDI	SDI
**Na^+^**						
29	26.5 ± 3.9a	26.6 ± 3.0a	29.4 ± 0.8a	32.4 ± 1.9a	32.2 ± 4.9a	34.1 ± 3.3a
91	22.3 ± 2.8a	19.5 ± 4.5a	14.4 ± 1.1a	28.5 ± 3.1a	24.8 ± 2.6a	26.4 ± 0.9a
120	25.0 ± 2.8a	19.6 ± 0.7a	23.2 ± 2.3a	35.2 ± 4.4a	34.3 ± 7.6a	35.5 ± 5.5a
147	30.3 ± 6.2a	18.2 ± 2.5a	18.2 ± 2.5a	44.6 ± 3.6a	37.2 ± 4.6a	33.3 ± 0.5a
174	18.9 ± 1.8a	20.8 ± 2.2a	15.7 ± 2.2a	41.5 ± 3.4a	30.9 ± 5.0a	33.7 ± 4.0a
202	17.8 ± 3.0a	16.9 ± 1.3a	17.8 ± 3.6a	32.7 ± 3.7a	40.5 ± 2.1a	37.6 ± 4.3a
**Cl^−^**						
29	40.3 ± 13.8a	52.6 ± 0.6a	60.5 ± 2.8a	59.6 ± 3.5a	61.9 ± 2.6a	50.2 ± 23.2a
91	84.9 ± 7.8a	89.7 ± 10.5a	51.9 ± 5.2b	54.4 ± 3.5a	80.6 ± 4.9b	76.7 ± 8.2b
120	64.9 ± 8.6a	73.1 ± 6.1a	47.4 ± 21.9a	81.6 ± 4.7a	64.7 ± 12.1a	59.6 ± 25.6a
147	77.9 ± 10.3a	68.3 ± 4.7a	68.3 ± 4.8a	73.9 ± 1.5a	50.6 ± 13.5a	97.4 ± 16.6a
174	96.2 ± 8.9a	56.7 ± 9.1a	70.0 ± 3.1a	81.7 ± 3.6a	72.9 ± 2.8a	76.1 ± 2.4a
202	67.45 ± 5.9a	68.6 ± 2.0a	59.6 ± 5.2a	66.9 ± 3.9a	83.4 ± 19.1a	68.3 ± 0.8a

## Data Availability

Not applicable.
